# Online peer to peer support: Qualitative analysis of UK and US open mental health Facebook groups

**DOI:** 10.1177/2055207620979209

**Published:** 2020-12-10

**Authors:** Julie Prescott, Amy Leigh Rathbone, Gill Brown

**Affiliations:** School of Education and Psychology, University of Bolton, Bolton, UK

**Keywords:** Online, peer, support, UK, US: mental health

## Abstract

**Objective:**

This research aimed to gain further understanding of how open Facebook groups are used for online peer to peer support and identify any similarities and/or differences between UK and US groups.

**Method:**

A systematic search of mental health related open Facebook groups was conducted using relevant key words. The posts from 14 UK and 11 US groups were acquired over a three month period and content thematically analysed using Nvivo.

**Results:**

Findings support previous research which evidences that online peer to peer support is beneficial for users seeking mental health information. Said support can increase feelings of connectedness, reduce feelings of isolation, and provide a platform for comparison of perspectives relating to personal experiences. Group membership may offer hope and increase feelings of empowerment in those using Facebook groups as a support mechanism. There was similar discourse seen throughout both UK and US posts in regards to gender inequality, lack of awareness and stigmatisation.

**Conclusions:**

The study highlights the positive impact of shared personal experiences, and offers a greater understanding of the benefits of online peer to peer support for mental health and wellbeing. There is evidence that, whilst mental health is becoming a more widely discussed topic, in both the UK and US, it remains negatively perceived. Questions are posed for group administrators and health professionals relating to their utilisation and moderation of such online peer to peer support networks.

## Introduction

### Online platforms for health

During 2017, 90% of all UK residential living had access to the World Wide Web, with almost three quarters of people (73%) accessing the internet using a tablet or mobile phone.^1,2^ Over the last decade, online social networking sites (SNS) have become more prevalent. Usage of SNS has increased tenfold between 2005 and 2015, with 69% of US adults,^3^ and 66% of UK adults using online social media platforms. Researchers are increasingly utilising SNS, such as Facebook and Twitter to obtain subjective health information and to recruit users for health related studies.^4,5^ In addition, recent years have seen an increase in the use of particular SNS; such as Facebook, Twitter and YouTube for personal health related information seeking and sharing, for both physical and mental health.^2,6–15^

### Online peer to peer support

Online peer to peer support (oPTPS) is using the online platform to assist or advocate for the self or others. This support can be delivered in many ways, such as; financially, emotionally, physically and socially. In contemporary society, oPTPS has become further engaged in via the online platform, and the use of SNS.^16–18^ Whilst individuals may lean towards oPTPS, its efficacy remains in refute due to both positive and negative aspects.^19,20^

Participating in oPTPS allows users to engage in the transference of informational and emotional support and share their experiences with others globally.^21^ oPTPS has repeatedly evidenced efficacy in eradicating feelings of loneliness, breaking barriers regarding stigma, promoting self-empowerment and encouraging individuals^22–24^ to become more active and informed patients. Individuals seeking oPTPS may believe the information they retrieve to be more trustworthy,^25^ be reassured by the ease of instantaneous access to their chosen online support network,^26^ reduce feelings of social isolation^27–29^ and grant a more secure level of anonymity for the support seeker.^30,31^

Despite the aforementioned benefits, due to the lack of social cues online, information may be misconstrued and misinterpreted, which, alongside anonymity, may instigate socially inappropriate responses.^32^ Concerns have been highlighted regarding information exchange; the absence of administrator moderation or professional input may lead to dissemination of incorrect information.^33,34^ There may also be incongruence between the perceived privacy of an online source and the actual level of privacy the site provides.^35^ Reliance upon and overuse of the internet for information, support and social communication may lead to internet addiction,^36^ resulting in a decrease in face-to-face interpersonal communication.^37^

### Online peer to peer support & social networking sites

oPTPS can be engaged with in several ways, for example; through websites, chat rooms and online mental health communities. One platform which has increased in popularity is social networking sites (SNS). For example, people who utilise YouTube may post informational videos and offer supportive comments, where after, likeminded people can reply, essentially offering and retrieving peer to peer support. The concept is similar for SNS such as Twitter. Users may post informational, emotional and supportive Tweets which other users can retweet or reply to. Facebook has developed to become, and remains, the most popular SNS universally.^38^

Users are increasingly turning to SNS as a source of informational and emotional support.^39^ Studies have found several positive benefits of using SNS for mental health issues, such as; an increase in perceived social support,^40^ feelings of empowerment and hope,^18^ reduced feelings of isolation,^40^ and a decrease in depressive symptoms.^18^ In addition, those seeking oPTPS have reported a lower level of stigmatisation,^41^ and increased feelings of connectedness.^42^ The efficacy of oPTPS via SNS has proven to be particularly prominent for women during pregnancy,^14^ and those with mental illnesses such as Schizophrenia,^43^ Psychosis^44^ and PTSD.^45^

Whilst considering the positive aspects, it is salient to address research which highlights some negative effects of using SNS such as Facebook in addition to the potential negative effects of general internet usage.^32–37^ Research has found that SNS have a negative impact on both work-related performance and happiness,^46^ whilst other studies suggest that Facebook use may lead to social comparison and envy,^47^ increasing the likelihood of negative self-perception in some people.^48^ More generally Facebook use may slow or impair stress recovery.^49^

Whilst using SNS for oPTPS could be deemed isolating due to lack of in person social interaction and confusing due to the transference of misinformation, the online platform is usable and efficacious for the most at risk individuals, such as men,^50,51^ young people^52–54^ and individuals from varying socioeconomic and ethnic backgrounds,^55,56^ and has the capacity to encourage conversation regarding mental health, essentially reducing the stigma which shrouds the topic.^57–59^

### Mental health in the UK and US

In terms of access to health care there are stark disparities between the UK and the US. From birth, those in the UK are automatically enrolled onto the national health care system (NHS)^60^ a complex assemblage of organisations which provide support and care to patients. The NHS is publicly funded^61^ and treatment is free at the point of service for all. In contrast, US healthcare is largely privatised, and the enrolment process for basic coverage is voluntary.^60^ The main source of health care cover is private employer based and/or individual insurance.^60^ Medicare and Medicaid are US government funded health plans designed for those who may face financial obstacles to accessing care, such as; the elderly, those on low income, underage individuals and those with disabilities.^62,63^ In the US access to health care for mental health issues can be impeded due to financial status. In 2018, 41.5% of US adults avoided accessing health care for mental health issues due to the fact that they could not afford the cost.^64,65^

Whilst the cost of accessing mental health care is not a foremost concern in the UK due to the NHS, consultant led referrals to mental health services can take anywhere up to 18 weeks.^66^ Also within the NHS, whilst choice is advocated where possible, patients surrender the right to choose which mental health service provider they want to use if they; require immediate care, already receive care for the condition, are a member of the armed forces and are young offenders, amongst other reasons.^66^ However, in the US, because health insurance is personally funded, access to care can essentially equate to consumerism; wherein patients can research their mental health care practitioners and choose providers, prior to meeting.

### Rationale & aim

The aim of this study is to gain further understanding of how open Facebook groups are used for peer to peer support and to identify any similarities and/or differences between UK and US groups.

Although research has been conducted comparing cultural differences in social media use between Western and East Asian societies,^67^ and regarding differences in general usage between five countries,^68^ there is no contemporary literature known to the authors which explores similarities and differences in the way people from two Western societies, the UK and US, use Facebook for oPTPS. Extant literature examining the use of online sources for oPTPS is extensive, however few studies have used qualitative research methods to analyse the use of Facebook for mental health support.

### Research questions

The research questions (RQs) for the study were as follows:

RQ1: How are open, mental-health related Facebook groups used for oPTPS in the UK & US?

RQ2: What type of oPTPS do users gain from open mental health related Facebook groups in the UK & US?

RQ3: Are there any similarities and/or differences between the discourse on UK and US open mental health related Facebook groups?

## Method

Authors elected to acquire data through Facebook as it is the largest used SNS and includes more open access oPTPS groups, specific to health issues, with rich qualitative data^69^; as opposed to other SNSs such as Twitter which is character restricted and Instagram which focuses more on pictures. There are Facebook groups increasing in numbers daily, specific to a variety of mental health issues, which are dedicated to empowering users and providing peer to peer support for those living with mental health issues.^18^ Most groups are monitored by one or more group administrator. These groups may be open or closed. In the closed groups, users must send a request to the group administrator to gain permission to join, whereas in open groups all users are free to join groups instantaneously. Facebook is an easily accessible platform for those who wish to seek and provide support to others using an online platform, disregarding country of origin. In the UK 66.2% of the population are active users of Facebook, as is 70.3% of the US.^70,71^

A systematic search of mental health related, open Facebook groups was conducted in January 2016 using mental health related keywords. The search terms were entered into the Facebook search bar. Only data from open groups were retrieved as permission to join these groups was not necessary to view content. The authors decided upon search terms which related to some of the most prevalent mental health issues and used the term mental health as it encompasses all terms. This search yielded a total of 154 Facebook groups; 38 by the keywords Mental Health, 38 Anxiety, 36 Depression, 16 Schizophrenia, 13 Stress, 8 Bipolar, and Psychosis yielded 5 results; as evidenced in Table 1.

**Table 1.** Key words searched and results yielded.

Search term	Number of results returned
Mental health	38
Anxiety	38
Depression	36
Schizophrenia	16
Stress	13
Bipolar	8
Psychosis	5

An Excel spread sheet was created with the Facebook group names, number of likes, the number of visits, the location of the administrator (where the group was set up/based) and the URL. Data from the groups (user comments) were copied and pasted into a word document for the purpose of this study. Each page consisted of individual user comments, which varied in length. It was subsequently converted into a PDF file. Each group was assigned a coded name, consisting of the file number, country of origin and date of collation.

### Inclusion criterion

Authors specified an inclusion criterion during the initial search on Facebook for relevant groups. Due to the specific aims of the study, only groups that had administrators based in the UK or US were considered for analysis. Only groups which used the English/American English language were included. Data were not included if it was not textual. This resulted in 26 UK based Facebook groups and 28 US based groups.

### Exclusion criterion

Authors devised a more stringent exclusion criterion which was applied to the groups. Groups were excluded from analysis if they consisted mainly of likes and shares with scarce user comments. These groups essentially lacked rich, qualitative data. Groups which displayed a lack of user engagement and were void of comments or posts over a two-month period prior to analysis were also excluded. Groups remained viable for analysis if they evidenced three month worth of qualitative data. This resulted in 14 UK Facebook groups and 11 US Facebook groups appropriate for analysis.

### Procedure

The PDF files for the remaining groups which were analysed were assigned new coded names comprised of a rank number between 1 and 25, based on the number of pages of data, and whether the data was from the UK or US, as portrayed in Table 2.

**Table 2.** Open Facebook groups (rank order based on number of pages of data).

Facebook group	Page topic	Number of pages	UK or US
01UK	Depression	523	UK
02US	Bipolar	515	US
03US	Depression and bipolar	344	US
04US	General mental health	332	US
05US	Anxiety and depression	284	US
06UK	Mental health charity	278	UK
07UK	Anxiety	174	UK
08UK	General mental health	168	UK
09UK	Stress (Veterans)	132	UK
10UK	General mental health	111	UK
11UK	General mental health	101	UK
12US	Mental health and substance abuse	101	US
13US	General mental health	100	US
14UK	Anxiety, depression and stress	72	UK
15US	Mental health intervention and training	69	US
16UK	Depression	69	UK
17UK	General mental health	66	UK
18UK	General mental health	60	UK
19UK	Bipolar	58	UK
20US	General mental health	57	US
21US	General mental health	53	US
22UK	General mental health support	52	UK
23UK	Anxiety	32	UK
24US	Anxiety	25	US
25US	General mental health	20	US

The data was collected from posts between 26/10/2015 to 26/01/2016 (or the closest date if there were no posts for that particular date). The resulting data set consisted of 1,901 pages of posts and comments from the 14 UK Facebook pages and from the 11 US Facebook, 1,900 pages of comments.

### Data analysis

In total, the overall data set consisted of 2801 pages of Facebook posts and comments. All data was analysed using QSR NVivo v.11.

**Figure 1.**
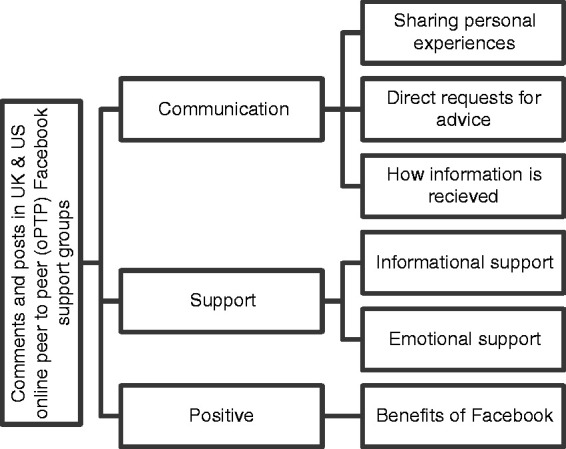
Final thematic map.

Authors followed the six phases, sequential process for reflexive thematic analysis as defined by Braun & Clark. In order to ascertain and increase validity of the analysis; data corpus was read by all authors.^72^ Next, initial codes were discussed, which organised data by number of pages and country of origin, generated and documented in a codebook. The codes were then used to generate themes and subsequent subthemes. The themes and subthemes generated were then reflected on in light of the data set to ensure that they shared an underpinning meaning. After reflection, the themes were refined, named and confirmed, as evidenced in Figure 1, which provides a visualisation of the final thematic map.

**Figure 2.**
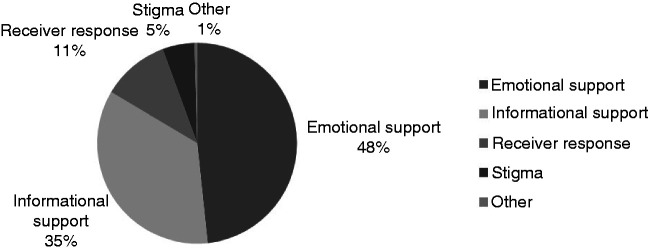
How social media is being used in relation to mental illness in the UK.

### Ethical approval and considerations

Approval for the study was granted by University of Bolton Research Ethics Committee in November 2015. Although the information used in this study was in the public domain it is acknowledged by the authors that posts were not intended for research purposes, and that no informed consent was gained.^73^ Quotes were gained via passive analysis and subjected to a Google search and a Facebook search to determine whether they could be traced back to the source. The searches yielded no results, however to further protect users’ privacy and minimise the risk of identification, no demographic data or possible identifying data was recorded and the names of the Facebook open groups were removed from this publication.^74^ In addition, some of the quotes were reduced in another attempt to limit the traceability of the text,^75^ and all quotes were made anonymous. As of yet, there is minimal guidance regarding the analysis of forum or group data. Due to the consideration needed for what actually constitutes as data within the public domain, and the fact that it could not be confirmed that all users were aware that they were posting and commenting in open groups, authors ensured that only open groups were used and all data remained anonymous to protect users.^76^

## Results

### Content analysis

In order to answer RQ1, inductive content analysis was used. The initial coded categories were derived directly from the contextual data^77^ and frequency of codes was recorded.^78^

In regards to the online user traffic within both US & UK groups, some were visited once where as others were visited up to 8756 times. The user ‘likes,’ of the groups as a whole, ranged from 53 to 231,129.

Of the UK data (see figure 2), 1358 (48%) references accounted for emotional support, such as; sharing experiences, inspirational quotes, encouragement and offers of support or further contact outside of the Facebook group; although some groups discouraged contact with others outside of the Facebook group. There were 989 references (35%) to informational support which consisted of requests for advice, signposting, information exchange and the encouragement to research validated health information. Accounting for the receivers response (11%) there were 305 references. Here receivers displayed gratitude, discussed getting help offline and fostered empowerment. There were 146 references to general stigma (5%). Comments and posts raised awareness, discussed the normalisation of symptoms and discussed community, personal, partner and family stigma experiences. The posts and comment labelled “other” mainly consisted of personal opinions during debates around mental health issues (1%); more specifically, the transition for veterans with PTSD into the way of civilian life Figure 1.

**Figure 3.**
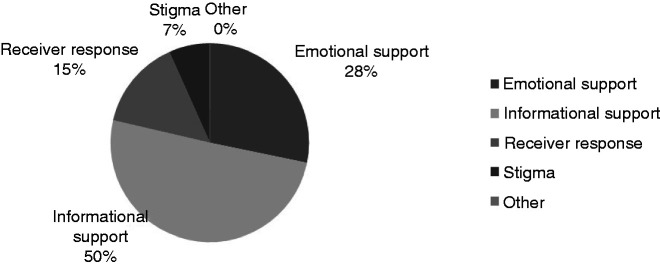
How social media is being used in relation to mental Illness in the US.

Overall, there were 4288 references. Of the US data, 1202 of the references accounted for emotional support. These also referred to sharing experiences, inspirational quotes, encouragement and offers of support or further contact outside of the Facebook group. In the US data, there was no discouragement of contact outside the realms of the groups. There were 2144 references (50%) to informational support which consisted of references to news articles, studies and petitions. Practical advice and signposting was also referenced, directing users to offline support, services and workshops. Medication and lifestyle changes were also discussed. Accounting for the receivers response (15%) there were 623 references Figure 3. Here receivers displayed gratitude for the groups’ peer to peer support discussed getting help offline and fostered empowerment. There were several posts that concerned politics which became heated. This data set concerned discussion of self-efficacy and changes in feelings and opinions. There were 284 references to general stigma (7%). Here the comments and posts were similar to the UK, in the sense that community, personal, partner and family stigma experiences were all discussed. There was only one post in the UK data which was categorised as “other.” This post spoke of medication being used by the government to terminate patients and directed users to a YouTube clip.

Whilst content analysis is beneficial when tallying frequency, it has been suggested that researchers risk eliminating implications of data as there is a danger of missing the context.^79,80^ Researchers have proposed that whilst the frequency of occurrence could suggest a greater topical importance within qualitative data, it could also suggest a greater disposition to discuss a particular topic.^80–82^ Considering the context of the data, that it concerns mental health issues and is in the public domain, the issue could be true for this study. To account for this the data set was next, thematically analysed. Inductive thematic analysis provides a more nuanced and complex account of the data.^83,84^

### Thematic analysis

Results yielded a range of 20-525 pages of comments and posts between the respective groups. The highest being a UK group for depression (n = 523) and a US group for bipolar (n = 515; refer to Table 1). The data had six main themes; Sharing Experiences, Informational Support, Emotional Support, Direct Requests for Advice, How Information is Received and Benefits of Social Media.

A range of issues were discussed which frequently reflected the specific focus and target group of the Facebook group such as anxiety or depression. However, within the more generic mental health groups, a range of mental health issues including anxiety, depression, stress, panic attacks, eating disorders, suicide, self-harm, substance misuse and postnatal depression were discussed. It was also evident from the discussions that mental health impacted all aspects of people’s lives including family, friendships, social aspects and employment. The quotes from users are presented below; participant number and location follow in brackets.

### Sharing personal experiences

One of the main features of the groups is that they allow people a place to share their experiences and stories. These stories came from personal experience, as well as family and friends affected by mental illness. This is true for both UK and US group.

Some of the US stories originated from those who had worked in mental health services. These users offered support referencing the lack of services and the stigma surrounding mental health. Although less frequently mentioned, this was also true of the UK data, as highlighted in the following quote from a retired mental health worker;

*It's only thanks to (FB group) and other groups that those suffering, carer's and society in general gets any real support and education. WE need to keep putting our views forward, support campaign groups, write to MPs and hold these people to account. (06UK)*

The data from both the UK and US evidenced conversations regarding the stigma that shrouds mental health issues. Both countries highlighted the pervasiveness of mental health stigma in very similar ways and it was often discussed using personal perception and experiences.

*I feel this isn't talked about enough. So much is said about depression and suicide, the presumption is that nearly everyone who has taken their life must have been suffering with depression. My 17-year-old son showed no more signs of depression than any other average teenager. I had no idea even considering his distress that his anxiety could drive him to make that fatal mistake. So here I am, it will be six months’ tomorrow and we are heading for our first Christmas without him. My heart is broken and my arms are empty. I wish I had have taken his anxiety a lot more seriously and realised what danger he was in. I miss you Sammy, I'm so very sorry baby. I love you. (05US)*

Stigma surrounding mental health was more frequently discussed in the UK groups. Media exposure and the role of celebrities were debated, evincing mixed opinions as to whether they helped or hindered mental health issues.

*I wish society would stop romanticising it. It's often a horrible, embarrassing, identity-scrambling and debilitating experience and we're not all bloody high functioning celebrities with private healthcare. (19UK)*

*The more people are talking about mental illness and bipolar disorder the more chance is that people will be more understanding and stop the stigma of mental illness all over the world, it's good that the story is on TV. (19UK)*

*I definitely do not recommend telling your employer. The day I told my employer that I had a mental illness that was the end of my career. One of the worst decisions of my life telling work about my mental illness. Can you imagine if Cancer patients were treated with the same dignity and compassion as the mentally ill. (04US)*

*Our son is disabled. We are isolated because we cannot enter the playground. My son has autism and a Hnf 1 beta gene which effects his speech and general manner. The looks we get also because my daughter has a stigmatization of her eyes. I had had kids laughing at her, the police have been involved. Disability discrimination is a crime but the police do also little…we are just a family trying to deal with the everyday challenges. (17UK)*

Gender differences in both the UK and US were often noted with users commenting about the lack of support available to men in regard to their mental health. The following quote illustrates gender differences in terms of communication style in terms of men not talking about their feelings.

*The thing is men don't talk about those issues cause they can be embarrassing where women seem to talk about near enough anything Men shouldn't be like that and I suppose other men should understand other men's feelings or we'll just have the stigma of this going crazy! (17UK)*

Not only did people share personal stories of their condition, but also their experience of subsequent healthcare. If people had struggled to receive what they perceived to be adequate healthcare, they became frustrated. The UK portrayed a negative discussion and attitude towards Cognitive Behavioural Therapy (CBT) whereas the therapy was not referred to in the US data. This evidences that there could be a difference in therapeutic approaches towards mental health between the two countries. In the UK CBT is the most widely available therapy provided by the National Health Service (NHS).

*Fingers crossed this will give people who haven't moved forward with CBT a feeling of hope. CBT doesn't work for everyone and hopefully the NHS will soon recognise this and start to fund other therapies! (18UK)*

*Sad part is good luck finding a good mental health physician. One let me diagnose myself because I worked in the medical field & asked what meds I wanted!! Another doped me up to being a zombie. I have actually found one now that I would follow anywhere. (12US)*

*10 month waiting for our first appointment with CAMHS for our daughter who is really struggling. I'm still waiting for a call back I rang again at 1pm today… Still waiting and getting angrier… If we'd have not turned up then that would have been it… No more appointment…(14UK)*

Alongside their experiences with and opinions of therapy, people shared their personal experiences of medications. The posts tended to be informative, including side effects. They also highlighted difficulties faced by those experiencing mental illness with regards to being unable to access the required medication and healthcare provisos.

*“Lithium worked well for me but I had to come off after 5 years becaus my white blood count went too high (which is one of the side effects) but it worked well.”(19UK)*

*I can't get Lortab for legitimate pain because of the high rate of abuse in (location removed). Keeping it away from kids is the parents job not the doctor's. (12US)*

### Direct requests for advice

People tended to openly request direct advice in all areas of mental health, such as; therapy, coping strategies and medication.

*I don't know. I'm depressed because I have nothing to motivate me. Does anyone here take Prozac? How long does it take to work? I've been taking it for a week now. (16UK)*

*Needing urgent help can't even go out nor look after myself with everything sorry (04US)*

Some also explored this avenue to receive information on how they can support others.

*My partner ticks all the boxes for bipolar we have been in and out of the GP for months now. It's so draining trying to fight for the right assessments can anyone who has experienced this help me with some advice on what I can do to push for this and be heard finally x (19UK)*

*How do I get help for my son and father within the guidelines of the law? They do not want help I'm feel like I'm going crazy dealing with all the highs and lows of Bipolar disorder and Schizo (04US)*

*My mum was diagnosed with bipolar after the death of my nana. Unfortunately, we haven't got it controlled yet. I'm now beginning to think about private healthcare? Or other possible options that might help her recover? But does anyone have any suggestions on what I can do to help my mum?? From first-hand experience or a carer?? (19UK)*

### How information is received

The support, advice and information provided via the comments were mainly received with, as one might expect, gratitude and appreciation. There were also a number of examples in both the UK and US data of people responding by not only thanking the person that provided the support but also by informing the person what action they planned to take based on the information provided.

*Thank you everyone for the help and encouragement you have given our son. Thank God for people like you, and all the staff at (location removed). xx.” (09UK)*

*Thank you. You are right. I do have my family here. I try not to let it bother me, but when the holidays roll around it's much harder. Thank you for listening to me and your words of encouragement. I will try hard to climb out of this, one step at a time. (03US)*

This adds to the support level as it can be observed that the information is valued and possibly also acted upon.

*Thank you for replying back to me, I am going to ring my CPN to see if she can get me an appointment with my psychiatrist. I will also ring your helpline in the meantime. I know I need the extra support now more than ever thank you again xxx.”*(06UK)

*Thank you, she gave me a number to contact someone in my city which I will get on to as I am very interested in finding out more about GRM. My other children are 19 to 29 so we might be a bit late for the book on how children grieve (smile emoticon)* (05US)

However, there were some instances of group members intervening when viewing a response, they do not feel is helpful or supportive as shown in the following quote, which suggests group cohesion.

*If you had you would know that it's not that simple. I'd like to add that using such a negative word as “sulking” to describe being depressed is hurtful and damaging. Perhaps you don't realise that the head of a person who's depressed is already full of self-critical, self-hating words and voices? The last thing they need is another…. (16UK)*

### Informational support

People tended to ask directly for informational advice on the groups, perhaps enabled and encouraged by the online platform due to accessibility and anonymity. Although groups are not officially moderated, there is evidence that administrators attempt to do so.

*I'm glad to see that no one responded to your request to message you. I have repeatedly said not to message with people until you have got to know them on the group. I hope you know what to do if someone shares very sensitive and personal information with you that could cause them to self-harm. I'm trying to keep you and the other people in my group safe. (01UK)*

The practical advice took the form of signposting to services, sharing web links, group information, and mental health related articles in the press, as shown in the following quote from the US data.

*If you feel like you’re experiencing something more pervasive than general sadness, make sure to see a physician before letting it get any worse… If you are depressed and want to seek treatment, there are plenty of websites, hotlines and other forms of professional help you can go to. Please do not wait to get the help you need! (04US)*

*I too am an ex submariner and have received invaluable help from (Facebook group). Just get in touch with the head office, they will do all they can to help. Take care mate. (09UK)*

*If you haven't already, check (Facebook group) on FB. Really great page. Super good video posted today by a young lady sharing her experience with bipolar. (04US)*

Sharing practical advice and possible helpful solutions that had worked for the individual commenting was also popular throughout the groups, such as the discussion to help reduce social anxiety.

Group administrators tended to offer informational support and encourage discussion within the groups, both in the UK and US. Groups also offered practical advice.

*If possible, expand your comfort zone in small steps. Attend a few small social events, such as drinks with colleagues after work or dinner with friends, and work up to larger social events. (07UK)*

*Look at all resources; get help from wherever you group. This group definitely helped my girlfriend and I. Local self-help groups are a great way of meeting people that know exactly where you are coming from I honestly hope anyone with this terrible disease find themselves on the road to recovery (12US)*

They also posted information around the group and any services it was involved in providing.

*Parent support group starts weds 9 Dec 5.30 to 7 at (location removed). Worried about your teen? Come along for a chat and a cuppa, we can also discuss best times and days to meet for future groups Drinks and refreshments provided! (14UK)*

*A community Care Station will be open for all interested January 21. Services will be available at (location removed) between the hours of 1:00 p.m. and 4:00 p.m., and again from 5:00 p.m. to 8:00 p.m. No appointment is necessary. (12US)*

Responses were similar in both the UK and US, with higher levels of engagement observed when quotes, pictures or articles were linked to current or relevant real-world events.

### Emotional support

The emotional support theme includes responses which were both empathetic and sympathetic. People tended to illustrate their comments with emoticons, although it is hard to decipher whether this was to express a deeper level of affection and support or a habitual response. Encouragement of personal experiences was rife using both directive and non-directive approaches. The non-directive approach involves people sharing their own similar experiences to provide encouragement and support whilst the directive approach involves people providing support and encouragement by simply wishing the person well with no personal disclosure or insight into their experiences offered.

*Because you have depression does not mean you are damaged. If someone has hurt you, I am sorry. (frown emoticon) Hugs (01UK)*

*Remember to look out for yourself! Engage in a self-care practice daily, and always look out for your needs first. That way you can fill up the cup with self-love and then give the leftovers to others. I have been engaging in a daily movement and breathing practice each morning and I feel as though it fills me with radiance, which I can then share with others. Do what you need to do! Much love (04US)*

What was interesting was frequent encouragement from someone with similar experiences and how they used personal disclosure of said experience to encourage and perhaps highlight the person was not alone.

*I'm so sorry to hear that (Anon). I too have suffered with anxiety for many years. It's so debilitating isn't it! At the moment, I seem to have it under control instead of it controlling me and its wonderful not to feel constantly worn out and running on empty. I'm telling you this so you know they're is light at the end of the tunnel and I sincerely hope you find it too. Wishing you peace of mind. Take lots of care and be kind to yourself. (Anon). (22UK)*

*Reach out to all those who can support you. I was suicidal in the first trimester also but you can pull through with the right help. It may not feel like it now but, there's always a light at the end of the tunnel. You're obviously very strong as you've beaten this before xxx (06UK)*

*I totally understand, sweetie. I lost my husband 34 years ago when I was pregnant with his only child. For years I got depressed around that time of year. I did something positive one year on my birthday and it helped to make the depression very mild. Try to find something very special to celebrate and it will help. Won't ever go away but perhaps you can be less depressed. Hope this is helpful to you. (05US)*

### Benefits of facebook

The benefits of social media were commented on in terms of the value of forums and the benefits of Facebook groups and the support they provide. Quotes imply the therapeutic benefits of commenting on Facebook groups and receiving supportive responses, highlighting the importance of SNS as a support mechanism.

*We all need to be heard! In the words of Frasire, “I am listening” and “good mental health” mean a lot more that poor advice.” (06UK)*

*Thank you (Facebook group) for all the hard work and kindness you show me and it makes me know that you’re in the journey with me and that I'm not alone and there is hope. (04US)*

*“I love these groups!! I stay on fb cause of y’all …” ““hugs always here if u want to talk. door is always open”” “hugs to all” (01UK)*

*I am looking for a group that accepts the mentally ill for who they are. A group that doesn't try to fix people. A group with at least a few of the administrators actually struggle with a mental illness themselves. A group that doesn't just promote long term medicine, but also maybe yoga, or meditation. A group that can find a support group in my area. A group that accepts me for me, on my good days, and my bad. (04US)*

Some people discuss the negatives of social media use and Facebook, such as; lack of face to face interaction, loneliness and boasting on Facebook which others can react negatively to. This is interesting since it is via Facebook that people are discussing issues and are ultimately connecting.

*Social media made everyone lonely…no one goes out to meet up with friends any more…they would rather sit in looking at Facebook on their smart phones….very sad…. (17UK)*

## Discussion

### Facebook support groups for mental health

In general, people communicate on Facebook open groups set up for mental health purposes to share their experiences of living with mental health and to seek oPTPS. The current study examined how people in the UK and US with a diagnosed or suspected mental illness or those who experience mental illness through those around them, utilized open Facebook groups in relation to mental health. Previous research suggests Facebook is a valuable platform for sharing personal experiences of mental illness and increasing the individuals’ perception of the level of social support they received^11,18^ in addition to support gained from family, friends and professionals.

Overall, use of Facebook groups was found to be friendly, supportive and informal, with many comments containing tagged names and emoticons to reinforce and personalize the posters message content.^14^ By sharing personal experiences of their mental illness, Facebook users provided informational and emotional support to other group members via oPTPS that may not be available from the receivers own social network or professionals, in line with previous research,^53,85^ oPTPS may lead to increased feelings of connectedness for the receiver,^41^ potentially reduce feelings of social isolation.^22,27–29^ This method of support acquisition may offer hope to people using Facebook for mental illness,^23^ thus empowering them to seek help further or take positive action.

In addition, users made direct requests for information and advice for both themselves and close family members on both the UK and US pages. The main topics of discussion related to medication and treatment, and group members tended to ask what other users would do if in a similar situation. Responses to comments tended to be empathetic yet constructive and informative, often offering practical advice and solutions, and again this was observed in both the UK and US data. Whilst some open Facebook groups are run locally, many targeted an international demographic, with groups allowing users from any country to join. This could reduce geographical constraints and provide access to hard-to-reach demographics,^50–52,55,56^ as individuals may join groups on a global scale. Indeed this could diminish social barriers often related to mental health care and support seeking, in particular feelings of stigmatisation.^58,59^ Thus, the diversity of group membership may have offered members alternative perspectives from individuals from differing countries and cultures when seeking support and advice, supporting previous findings.^86^

Individuals may be more inclined to trust information gained from such an extensive pool of information, however this may limit the ability to signpost to appropriate offline services. Having some trust that the information received is accurate may have promoted autonomy in the individual regarding choosing a course of action. Facebook users in receipt of advice and information often expressed their gratitude toward both peers and pages, discussing their intended actions in relation to mental health and wellbeing. This suggests that advice given may be followed, again highlighting the importance of providing accurate information,^2^ and the importance of moderation when information is sought online.^53^

In contrast to prior research surrounding the negative impact of using SNS for information and support,^41–44^ this study found that people using open Facebook groups felt, on the whole, that the support they received from online peers had a positive impact on feelings of empowerment and hope,^23^ their perceived social support network,^22^ and feelings of stigmatization were reduced.^24^ Based on the observable data from both the UK and the US, stigma relating to mental illness was shown to be a pervasive factor in people’s lives, and both data sets highlighted the following points. Whilst opinion regarding how the media and celebrities portray mental illness was often conflicting, the general consensus from Facebook group users in this study was that there is a need for more open discussion relating to mental health to raise awareness and accurate information.^21^

It is uncertain as to whether the administrators of the groups were mental health professionals. This data could not be ascertained without direct questioning which did not occur in the passive analysis. However, ensuring that the correct information is available and eliminating stigma within the groups was found to be of great importance to people from both the UK and US, and moderation of user posts was conducted by both administrators and other group users. This was particularly evident when examining how users received comments on fellow group member’s posts. Often the disclosures from group members were detailed and offered a lot of personal information, similar to recent findings relating to online blogs indicating that the poster felt that they were in a safe place to discuss their experiences. This is indicative of a sense of group cohesion, and is supportive of previous research findings.^53,57^

However, there were instances of inappropriate responses to comments. This could be due to several reasons. For example, the person may not have proof read their response, there may have been a lack of awareness of and reflection upon how their response would be perceived by others or it could possibly be due to disinhibition.^32^ Regardless of the reason for an inappropriate response, they could have a negative effect on the original poster. Group members were quick to respond to comments which appeared to promote misinformation and stigmatization in defence of the original poster. Whilst this is a negative outcome of using Facebook for mental health support, the only regulatory bodies appointed on this SNS is the administrator. As much as the administrator of the group can advise on issues and moderate the comments, if a group is of a substantial size then there is a possibility that debilitating comments may go unaddressed, leaving the original poster vulnerable to personal opinions and responses from others which may entail negative connotation. Again, this was true for both the UK and US data. Group members and administrators on one particular page were united in their rebuttal of a group member’s comments suggesting that the symptoms of depression could be described as ‘sulking.’ This is supportive of the notion of cohesion within the group^58^ possibly increasing feelings of connectedness,^86^ and perceived social support.^18,22^

### Mental health in the UK and US

Pages were analysed from both the UK and US Facebook pages to ascertain differences and similarities in users’ approaches, needs and experiences. When exploring the differences and similarities between the UK and the US in regards to how they retrieve support surrounding the topic of mental health via Facebook, many similarities were evident. There was similar discourse from both the UK and US that pertained to gender inequality and a lack of awareness of mental health issues.

Those in the UK and US purported to have similar experiences with stigmatisation in regards to mental health. Individuals must strive to conquer not only personal barriers to support seeking, such as personal attitudes, poor mental health literacy and mental health care avoidant type behaviour, but also provider and system-level barriers, which is inclusive of, but not exclusive to, financial constraints and lack of insurance.^58^ This is interesting that each barrier to be overcome by each country seem to entail a particular hindrance.

Access to the NHS is free at the point of treatment and furthermore so in the UK. This is also true for mental health care, with counselling offered on the NHS. There is an option to enrol in self-funded, private care if a patient wishes. Whilst patients in the UK have access to a proviso such as the NHS, there remains an underlying cultural stigma regarding therapy and mental health issues.^62,87–93^ Conversely, in the US mental health seems to be a more discussed topic, yet their access to mental health care may only be facilitated by insurance packages and adequate funding.^60,94,95^ It is possible that it is for these reasons that both countries may face barriers to overcoming stigma around mental health issues. Whilst one is cultural and another is financial, due to these reasons, stigmatisation and barriers to accessing services remains a concern across both the UK and US.^61,63–65^

From the findings, it was noted that comments from the UK Facebook data tended to show a slightly higher frequency of self-disclosure in general. However, people in the US tended to further disclose their own personal experiences when commenting on current articles and news stories relating to mental health. Referring back to the aforementioned issue of stigmatisation, people from the UK may possibly disclose more on Facebook as the people within this group are strangers, as opposed to people they come into contact with in day to day life. The online platform grants a certain level of anonymity and people may feel as though they are not being judged by others. People from the US may disclose more online when discussing current articles and news stories as they feel that the publicity and expose are working towards eradicating stigma shrouding mental health issues in public domain.

In the US, attending therapy sessions for mental health issues is a more normative practise than in the UK. This could explain why therapy was not referred to in the US groups. In the US, those with health insurance can choose their own therapist, essentially playing the role of an active patient by choosing which therapist and therapy they would prefer to undertake. In the UK, CBT is the initial therapy which is offered to anyone in need on the NHS. If CBT is not an effective therapy for the patient and said patient cannot afford a private therapist, this can leading to, not only negative opinions of CBT, but also therapy as a whole.

Overall, when exploring the UK and US groups comparatively, it was initially apparent that the majority of both groups sought informational and emotional support from social media in regards to their mental health. Regardless of country of origin, users are facing fewer obstacles to peer to peer support by using the internet as a medium for connection. Both the US and UK still face stigma in regards to their mental health, yet interestingly, self-disclosure of mental health issues was more prevalent in UK groups. This poses the question as to whether it is due to therapist use being greater in the UK. Both groups also noted issues surrounding gender equality. It can be suggested that the more that users from both countries discuss their mental health online and engage in self-disclosure, then stigma may be minimised on a global scale.

### Strengths and limitations

Despite the growing body of literature relating to the use of online sources for informational and emotional support, little research is available which focuses solely on the use of Facebook as a support mechanism for people seeking support surrounding mental illness. The current study utilized a large, rich data set of online interactions in open Facebook groups, and has provided a novel insight into how group members interact and support each other online. A deeper understanding of how non-directional support is provided through relating personal stories and experiences has been gained, and how both group members and administrators use directional and non-directional approaches to impart information and gain advice.^53^

A particular strength of the study is that both the posts and comments, and the receiver responses were available for analysis. Receiver responses were openly available for analysis, and provided a depth of insight into how group members reacted to differing types of posts and comments, and also whether any action was intended in response to advice given. Unless posts and comments are removed by the poster or group administrator, they are available to read indefinitely, and therefore open to additional responses and the evolution of discussions over time.

One salient limitation is that there was no way to ascertain whether members of the group had a clinical diagnosis of mental health, due to the nature and methodology of the study. Whilst many of the group’s members claimed to have mental health issues, the authors were not aware whether these diagnoses were self-imposed. It is plausible that some members used the groups to gain support if they were surrounded by others with mental health issues. Due to the nature of the groups, no demographic data is obtainable nor is any information about the mental health of the people who seek or indeed provide support via Facebook.

The data set is limited to a set time period, which for this study was three months. It may be interesting to understand if people use online resources such as Facebook during particular periods of time or perhaps when a media article highlights an issue. Also, no data relating to whether the members of open Facebook groups sought support elsewhere, such as from professional services, was observed. Some groups specified age restrictions, therefore it may be assumed group members were adults; however platform providers cannot, and do not, deter younger people who do not meet the age criteria from adjusting their personal profile details, such as their date of birth. Therefore, the results of the study cannot be generalised to a wider demographic.

### Future research

Future research might explore in more depth the attitudes and opinions of those Facebook users who view using Facebook negatively in terms of reducing face-to-face contact and increasing feelings of loneliness. Furthermore, additional research might explore how individuals use Facebook the mental health support groups for oPTPS in conjunction with any other online or offline mental health services. Additionally, utilization of oPTPS sought from closed Facebook groups could be explored and comparisons made with open groups to examine differences in moderation style, levels of disclosure, and overall engagement. This raises several implications for UK and US group administrators, moderators, health professionals and service providers, in that possessing a deeper understanding of the way people are using Facebook could inform the way groups are administrated in the future and it may help direct the development of professionally run online services. Future analysis of closed Facebook groups with higher levels of privacy may provide an even deeper understanding of how these groups are utilised for mental health support purposes. Further exploration of such demographics would provide a greater insight into who is using Facebook and how they are using it as a support mechanism. This information could highlight valuable information relating to demographics which do not use open Facebook groups, providing useful information for administrators, professionals and service providers regarding hard to reach populations.

## Conclusions

This study adds to the current growing body of research examining how people with mental illness access oPTPS, and provides a unique and in-depth insight into how people with mental illness use open Facebook groups specifically to seek and provide emotional and informational support online. The use of Facebook for support with mental illness was similar across both the UK and US, with both societies highlighting the same issues such as stigma, lack of awareness, and barriers to accessing services. However, there were some differences in regard to possible reasons for stigma and attitudes towards therapy. Moreover, the study highlights the positive impact of shared personal experiences, and offers a greater understanding of the benefits of online peer support in relation to mental health and wellbeing. There is a clear requirement for further research into how people are using open Facebook mental health groups, particularly in relation to the demographics of users, and questions are raised for group administrators and health professionals regarding how they can best moderate and utilise the online Facebook platform to provide a safe environment and accurate information.
